# Genome characteristics of the *optrA*-positive *Clostridium perfringens* strain QHY-2 carrying a novel plasmid type

**DOI:** 10.1128/msystems.00535-23

**Published:** 2023-07-17

**Authors:** Ke Wu, Zhe Li, Mingjin Fang, Yuan Yuan, Edward M. Fox, Yingqiu Liu, Ruichao Li, Li Bai, Wen Zhang, Wei-Min Zhang, Qi Yang, Lingling Chang, Pu Li, Xinglong Wang, Juan Wang, Zengqi Yang

**Affiliations:** 1 Department of Preventive Veterinary Medicine, College of Veterinary Medicine, Northwest A&F University, Yangling, China; 2 Key Laboratory for Prevention and Control of Major Ruminant Diseases, Ministry of Agriculture and Rural Affairs, Yangling, China; 3 Bureau of Agriculture and Rural Affairs, Junan, China; 4 Department of Applied Sciences, Northumbria University, Newcastle upon Tyne, United Kingdom; 5 Department of Basic Veterinary Medicine, College of Veterinary Medicine, Yangzhou University, Yangzhou, China; 6 Research Unit of Food Safety, Chinese Academy of Medical Sciences (No. 2019RU014); NHC Key Lab of Food Safety Risk Assessment, China National Center for Food Safety Risk Assessment (CFSA), Beijing, China; 7 Ningxia Supervision Institute for Veterinary Drugs and Animal Feedstuffs, Yinchuan, Ningxia, China; 8 Department of Critical Care Medicine, the Second Affiliated Hospital of Air Force Medical University, Shaanxi, China; Pacific Northwest National Laboratory, Richland, Washington, DC, USA

**Keywords:** *Clostridium perfringens*, plasmid, *optrA*, Tn*6218 *transposon

## Abstract

*Clostridium perfringens* is a bacterial species of importance to both public and animal health. The gene *optrA* is the first gene that confers resistance to the tedizolid, a last-resort antimicrobial agent in human medicine. Herein, we whole-genome sequenced and analyzed one *optrA*-positive *C. perfringens* strain QHY-2 from Tibetan sheep in Qinghai province and identified one *optrA* plasmid pQHY-2. The plasmid shared similar structure with the *optrA*-positive plasmids p2C45 and p21-D-5b previously identified in *C. perfringens*, demonstrating the potential horizontal transmission of the *optrA* plasmids among *C. perfringens* strains. Annotation of the *optrA*-positive plasmids showed *optrA* and *erm*(A) located on a segment flanked by IS element IS*1216E*, and *fexA*, *optrA,* and *erm*(A) located on a segment flanked by IS element IS*Vlu1*, which revealed the possible dissemination mechanism. Additionally, a Tn*6218*-like transposon carrying *aac(6′)-aph(2″*) and *erm*(B) was also detected on pQHY-2, demonstrating the transposition of Tn*6218* and spread of antibiotic resistance among *Clostridium* bacteria. Molecular analysis indicated the *optrA*-positive plasmids belonged to a plasmid type distinct from the pCW3-like plasmids, pCP13-like plasmids, or pIP404-like plasmids. Further structure analysis showed they might be formed by inserting segments into plasmid pCPCPI53k-r1_1, which coexist with two pCW3-like plasmids and one pCP13-like plasmid in *C. perfringens* strain CPI 53k-r1 isolated from a healthy human in Finland.

**IMPORTANCE:**

Antimicrobial resistance is now a global concern posing threats to food safety and public health. The pCW3-like plasmids can encode several main toxin genes and three antibiotic resistance genes (ARGs), including *tetA*(P), *tetB*(P), and *erm*(B), which used to be considered as the main carrier of ARGs in *Clostridium perfringens*. In this study, we found the *optrA* plasmids, which belonged to a novel plasmid type, could also harbor many other ARGs, indicating this type of plasmid might be the potential repository of ARGs in *C. perfringens*. Additionally, this type of plasmid could coexist with the pCW3-like plasmids and pCP13-like plasmids that encoded toxin genes associated with gastrointestinal diseases, which showed the potential threat to public health.

## INTRODUCTION

Antimicrobial resistance (AMR) is currently one of the important threats to global health as well as food safety (1). The oxazolidinones are last-resort antimicrobial agents used for the treatment of severe infections in humans caused by multidrug-resistant (MDR) Gram-positive bacteria. Linezolid, the first fully synthesized oxazolidinone antibiotic, was approved by the US Food and Drug Administration for clinical use in 2000 (2). However, linezolid-resistant *Enterococcus* and *Staphylococcus* isolates have been reported since the year 2000 (3). The first reported linezolid resistance gene, *cfr*, encodes an RNA methyltransferase that modifies the adenine residue at position 2,503 of the 23S rRNA gene and thereby confers combined resistance to phenicols, lincosamides, linezolid, pleuromutilins, and streptogramin A antibiotics (4).

The OptrA protein, an ABC-F protein encoded by the *optrA* gene, mediates resistance against phenicols and oxazolidinones, by protecting the ribosomes of the bacteria. Compared to *cfr* gene, *optrA* can not only mediate resistance against linezolid but also tedizolid (5). *Clostridium perfringens* is a Gram-positive, anaerobic, rod-shaped bacterium, which is widely disseminated in natural environments (6). As an opportunistic pathogen, *C. perfringens* may be present in the intestinal tract of humans and animals, without causing disease (7). When the intestinal environment is disturbed, *C. perfringens* can multiply rapidly and secrete over 20 protein toxins, presenting a huge threat to livestock breeding and human public health and safety (8). *C. perfringens* can cause various animal gastrointestinal diseases, including necrotizing enteritis and diarrhea, which lead to huge economic losses in livestock breeding each year (9, 10). Moreover, *C. perfringens* strains can transmit from animals to humans through the food chain. Food poisoning caused by *C. perfringens* is one of the leading foodborne illnesses globally, being the second most common cause of foodborne illness outbreaks in the USA and South Korea, and the third most common in the UK and France (11).

Plasmids are important for the virulence of *Clostridial* species (12). Based on the type of replication initiator, the plasmids of *C. perfringens* are categorized into three broad families, consisting of the pCW3-like plasmids, pCP13-like plasmids, and pIP404-like plasmids. The size of pCW3-like plasmids varies from ~45 kb to ~135 kb, while the size of pCP13-like plasmids varies from ~36 kb to ~58 kb (13). However, the size of pIP404-like plasmids is smaller (~10 kb), and the plasmid structure is relatively simple (14). It is reported that the pCW3-like plasmids can harbor various toxin genes including β-toxin coding gene *cpb*, ɛ-toxin coding gene *etx*, and *C. perfringens* enterotoxin coding gene *cpe* (15, 16). Moreover, some antibiotic resistance genes (ARGs) [*tetA*(P), *tetB*(P), and *erm*(B)] can also locate on the pCW3-like plasmids (17, 18), which may lead to the co-spread of toxin genes and ARGs. The transfer of pCW3-like plasmids is based on the Tcp locus, composed of origin of transfer (*oriT*), *tcpA* to *tcpJ*, *tcpM*, and *tcpK* (19). Compared with pCW3-like plasmids, less toxin genes (*cons.cpb2*, *becA*, and *becB*) are known to locate on the pCP13-like plasmids, and no ARGs have been detected on them (20). The transfer of pCP13-like plasmids is based on the Pcp locus, an ~2.7-kb sequence segment consisting of *pcpA* to *pcpT* (21).

This study aimed to provide insights into the genetic characteristics of the *optrA* gene and the *optrA* plasmids in *C. perfringens*, as well as the genomic content of *C. perfringens* that harbor these plasmids. Herein, we whole-genome sequenced and analyzed the *optr*-positive *C. perfringens* strain QHY-2 isolated from Tibetan sheep in Qinghai province, compared it with other strains, including those harboring similar plasmids, and further investigated the structure and transferability of the *optrA* plasmids.

## RESULTS

### WGS analysis

After polymerase chain reaction (PCR) assays, agarose gel electrophoresis, and sequence alignment, QHY-2 was found to be positive for both phenicols-resistant gene *fexA* and oxazolidinones/phenicols-resistant gene *optrA*. Besides florfenicol, QHY-2 also showed strong resistance to chloramphenicol (MIC >64 µg/mL) and linezolid (MIC = 32 µg/mL). Whole-genome sequencing (WGS; NCBI BioSample: SAMN33306092) resulted in a total length of 3,528,159 bp, consisting of the chromosome sequence (3,361,707 bp) and an *optrA*-positive plasmid (166,452 bp, designated as pQHY-2). Eight types of IS elements were detected in the genome, where IS*Cpe5*, IS*Cpe2*, IS*1470*, and IS*Cpe4* were detected on the chromosome, while IS*Vlu1*, IS*256*, IS*1216E*, and IS*Cldi2* were detected on plasmid pQHY-2. Nine ARGs were also detected in QHY-2, in which *optrA*, *fexA*, aminoglycoside-resistant genes *aac(6′)-aph(2*″), and lincosamide/macrolide-resistant genes *erm*(Q), *erm*(B), and *erm*(A) located on plasmid pQHY-2, while aminoglycoside-resistant genes *ant (6)-Ib* and tetracycline-resistant genes *tet* (22) and *tetA*(P) were identified on the chromosome. In addition, 10 toxin genes, including phospholipase C coding gene *plc*, alpha-clostripain coding gene *cloSI*, perfringolysin O coding gene *pfoA*, collagenase coding gene *colA*, hyaluronidase coding genes *nagH*, *nagI*, and *nagK*, and exo-alpha-sialidase coding genes *nanI* and *nanJ,* were also detected on the chromosome.

**Fig 1:**
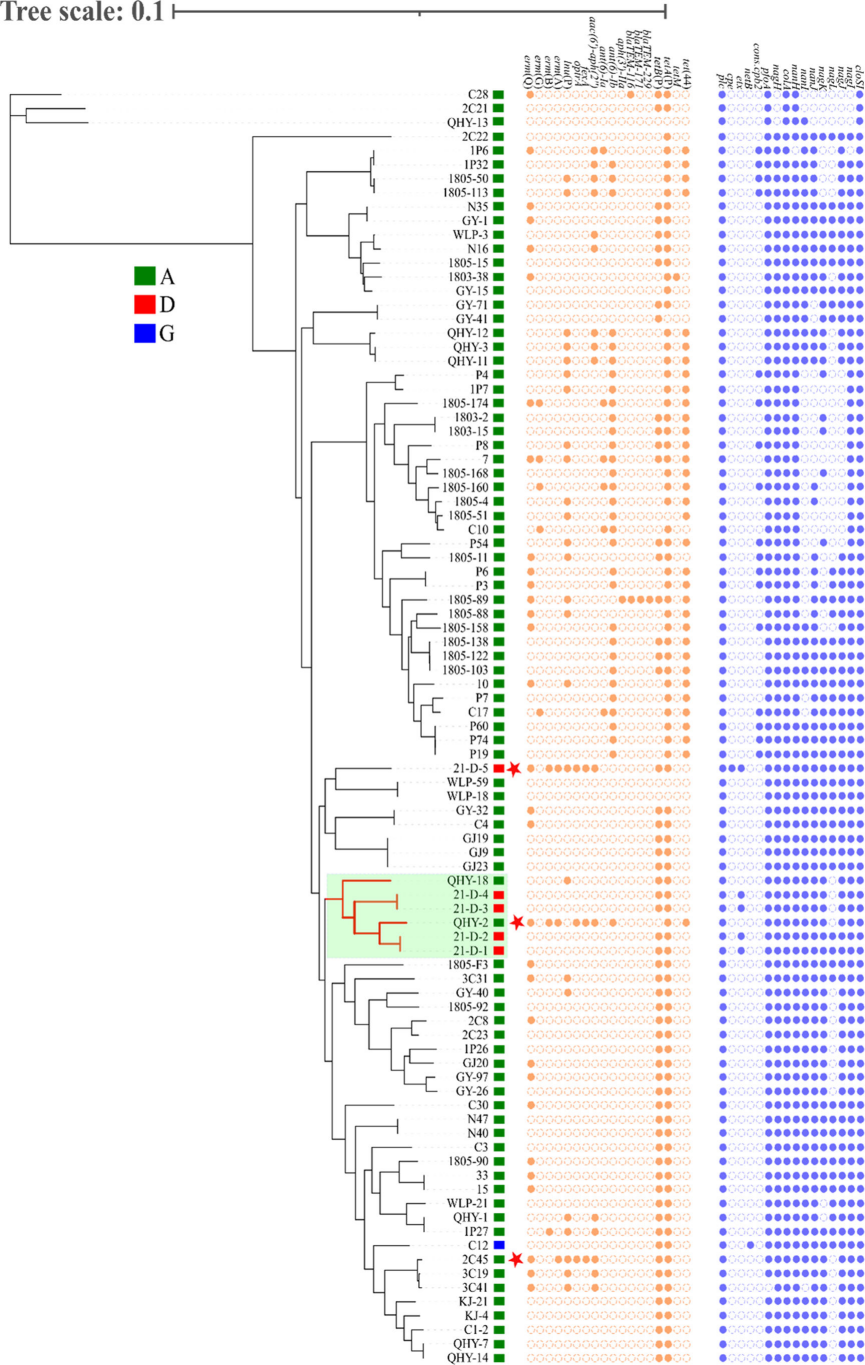
Phylogenetic tree based on the SNPs of the 91 *C*. *perfringens* strains from food animals in China. The branch in which QHY-2 locates on is marked with green shadow. The three *optrA* positive *C. perfringens* strains were marked with red stars. *C. perfringens* type A, D, and G strains were marked with green, red, and blue squares, respectively. SNP, single-nucleotide polymorphism.

Toxin typing indicated most of the *C. perfringens* isolates recovered from food animals in China belonged to *C. perfringens* type A, and many toxin genes, including the *cpe* gene linked to human foodborne gastroenteritis, were detected in them. In the phylogenetic tree, QHY-2 clustered together with another Tibetan sheep *C. perfringens* type A strain, QHY-18, from Qinghai province and four goat *C. perfringens* type D strains (21-D-1, 21-D-2, 21-D-3, and 21-D-4) from Shaanxi province (Fig. 1). Interestingly, the another *optrA*-positive *C. perfringens* type D strain (21-D-5) isolated from the same source as the strains 21-D-1, 21-D-2, 21-D-3, and 21-D-4, did not cluster within this clade, showing a certain genetic diversity of *C. perfringens* from the same source. The *C. perfringens* isolates from pigs appeared to be distinct from the other isolates in the cluster heatmap based on toxin gene profiles, particularly clade I, which was mainly composed of the *C. perfringens* isolates from pigs (Fig. S1). Moreover, 94.1% (16/17) of the beta-2 toxin coding gene *con.cpb2* was detected in the *C. perfringens* isolates from pigs (Fig. S2), suggesting that the porcine species might be an important reservoir of *C. perfringens* isolates containing this virulence marker.

### Structure of the *optrA* plasmids

**Fig 2:**
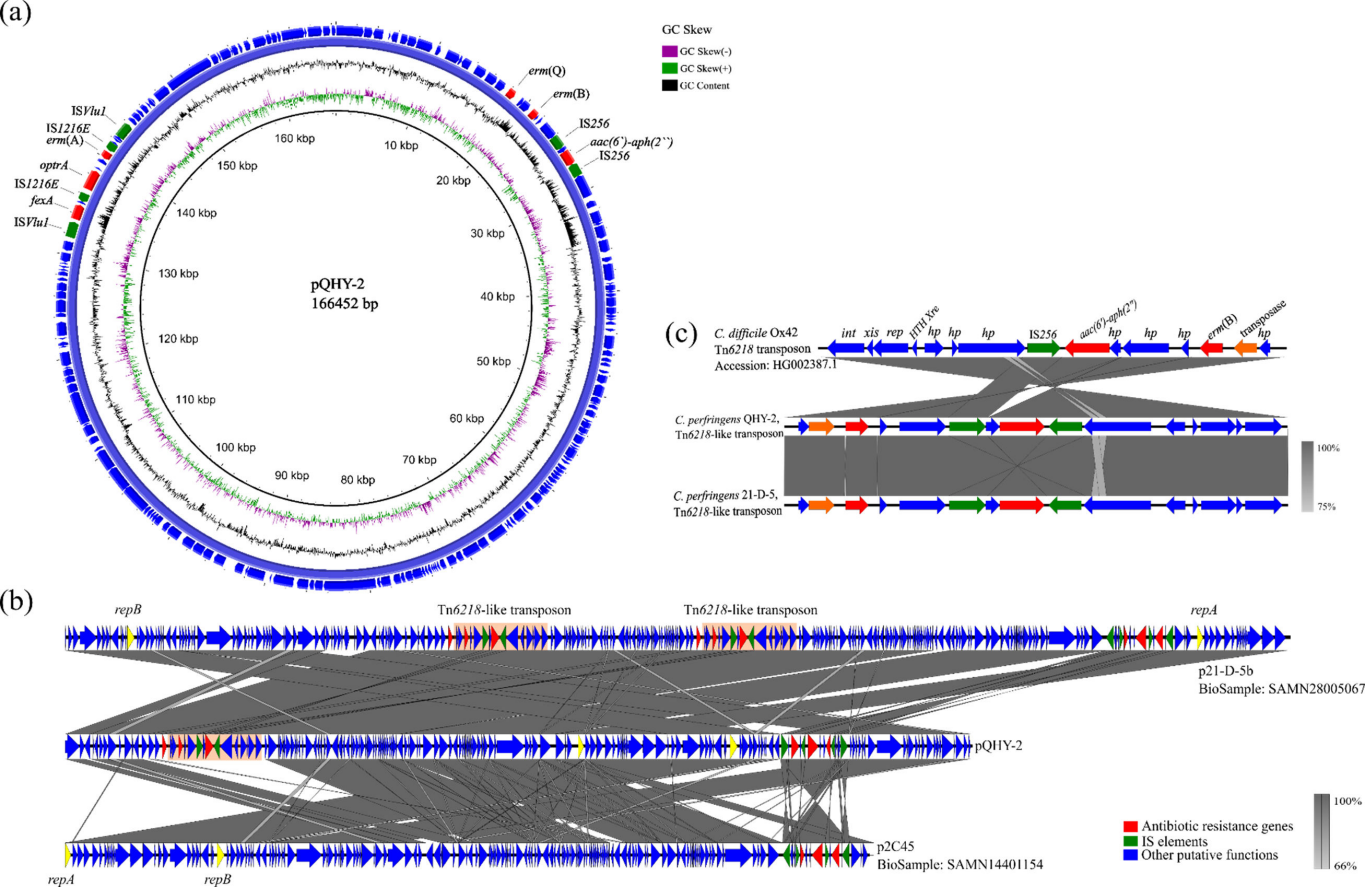
Character of the *optrA*-positive plasmids. (**a**) Structure of plasmid pQHY-2. (**b**) Sequence alignments among plasmids p2C45, pQHY-2, and p21-D-5b. Arrows indicate the directions of transcription of the genes. Tn*6218*-like transposons on plasmids pQHY-2 and p21-D-5b were marked with pink shadow. (**c**) Sequence analysis of Tn*6218* transposon in *C. difficile* Ox42 and *C. perfringens* pQHY-2 and p21-D-5b. Arrows indicate the directions of transcription of the genes.

The *optrA* gene on plasmid pQHY-2 (Fig. 2a) shared a 99.9% sequence identity with the *optrA* gene initially detected on *Enterococcus faecalis* plasmid pE349 (GenBank accession number KP399637) (23). The only polymorphism occurred at base 1,387 (from A to C), which leaded to the change of amino acid at position 463 from threonine to proline. Moreover, the respective segments containing *optrA* and *fexA* were both flanked by IS*Vlu1* on plasmids pE394 and pQHY-2, indicating the possible horizontal transfer of *optrA* and *fexA* from *E. faecalis* to *C. perfringens*, *vice versa*. Sequence alignments between pQHY-2 and the other two *optrA*-positive plasmids (p2C45 and p21-D-5b) previously reported in *C. perfringens* showed pQHY-2, p2C45, and p21-D-5b shared similar plasmid structure and high sequence identity (Fig. 2b), demonstrating the possible transmission of the *optrA* plasmids among *C. perfringens* isolates (24, 25). Some genetic elements that were homologous to parts of the clade 3 pathogenicity locus (PaLoc) insertion in *C. difficile* were assigned the designation Tn*6218*, and Tn*6218* transposons were mainly detected in *C. difficile* strains (26, 27). In this study, one Tn*6218*-like transposon that shared high sequence identities with the Tn*6218* transposon in *C. difficile* strain Ox42 (BioSample: SAMEA2052297) and the Tn*6218*-like transposon in *C. perfringens* strain 21-D-5 (BioSample: SAMN26351020) was detected on plasmid pQHY-2. Additionally, ARGs *aac(6′)-aph(2*″) and *erm*(B) were detected on the transposons (Fig. 2c), indicating the dissemination of Tn*6218* and associated antibiotic resistance among *Clostridium* bacteria.

### Phylogenetic analysis of the *optrA* plasmids

**Fig 3:**
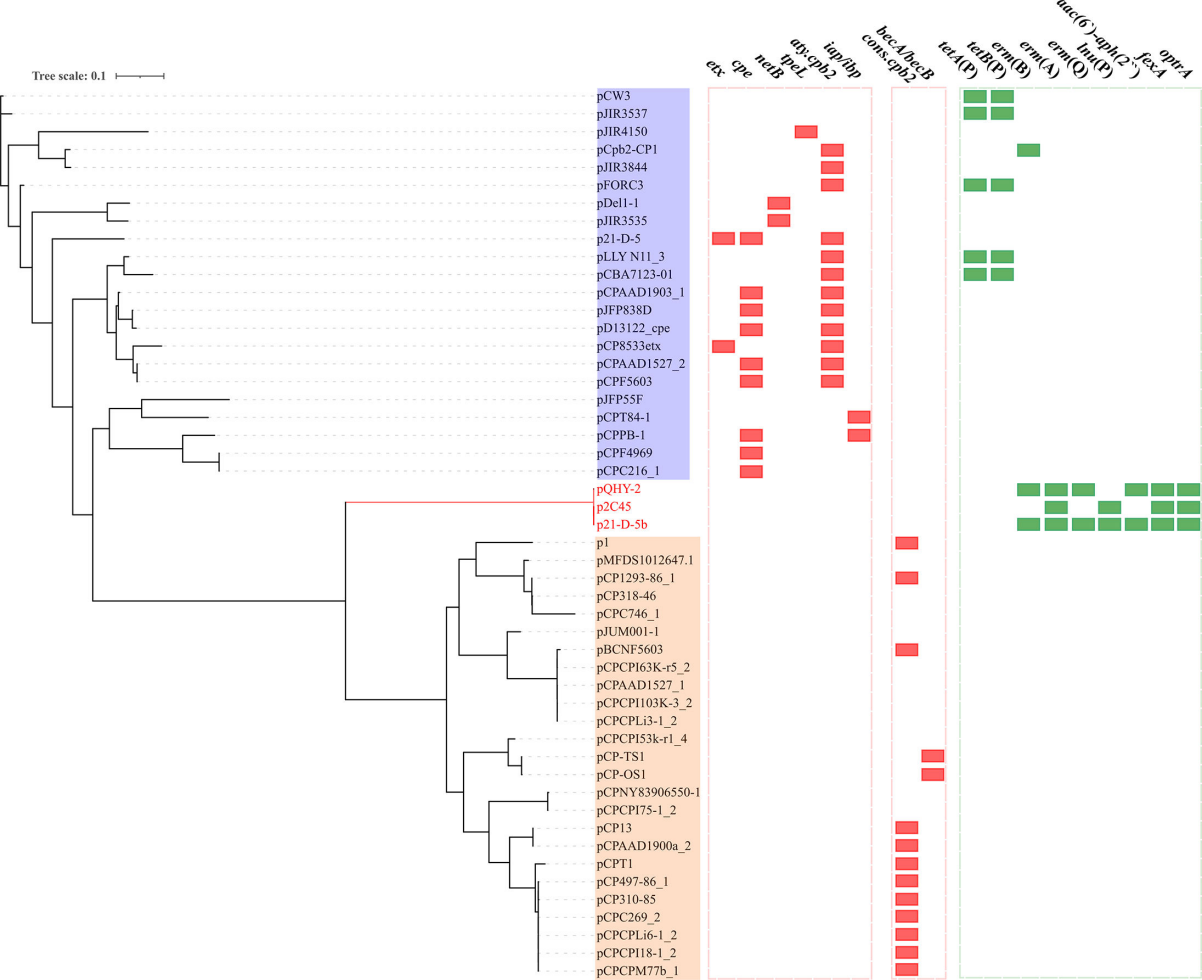
Phylogenetic tree of the *C. perfringens* plasmids. The pCW3-like plasmids and pCP13-like plasmids were marked with blue and orange shadows, respectively. The branch where *optrA*-positive plasmids (p2C45, p21-D-5, and pQHY-2) locate on was marked with red line. Toxin genes and ARGs were marked with red and green squares, respectively.

A previous study indicated that the *optrA* plasmid p2C45 could not be conjugated (25). In our study, both conjugation and electrotrans-formation experiments using QHY-2 and 21-D-5 as donor and *C. perfringens* isolates with low MICs (<8 µg/mL) to florfenicol showed unsuccessful transfer of pQHY-2 and p21-D-5b. To investigate the molecular mechanisms of these *optrA*-positive plasmids, we performed sequence alignment on the replication initiator coding genes and phylogenetic analysis. These *optrA*-positive plasmids all harbored two replication initiator coding genes, designated as *repA* and *repB* (Fig. 2c). Genes *repA* and *repB* on the *optrA* plasmids shared 100% sequence identity, respectively, indicating they belonged to the same plasmid type. However, *repA* and *repB* were not detectable on the pCW3-like plasmids, pCP13-like plasmids, or the smaller pIP404-like plasmids (Fig. S3). Then, a total of 50 *C*. *perfringens* plasmids (Table 1) consisting of the 3 *optrA* plasmids, 22 pCW3-like plasmids, and 25 pCP13-like plasmids were selected to construct a phylogenetic tree, using MAFFT and PHYLIP. Various toxin genes (*cpe*, *etx*, *netB*, etc.) and some ARGs including *tetA*(P), *tetB*(P), and *erm*(B) were detected on the pCW3-like plasmids, while three toxin genes, including *cons.cpb2* and the binary enterotoxin BEC coding genes *becA* and *becB* (28), were detectable on the pCP13-like plasmids. Compared to the pCW3-like plasmids and pCP13-like plasmids, the *optrA* plasmids harbored more ARGs, but no toxin genes, and neither the Tcp conjugal transfer locus nor Pcp conjugal transfer locus was detected on them. Moreover, the phylogenetic tree showed the *optrA*-positive plasmids clustered together on their own distinct clade, apart from the pCW3-like plasmids and pCP13-like plasmids (Fig. 3), indicating the *optrA*-positive plasmids belonged to a novel type of plasmid that differed from the smaller pIP404-like plasmids and the transferable pCW3-like plasmids and pCP13-like plasmids.

**TABLE 1:** Information of the plasmids used to construct the phylogenetic tree

Type	Plasmids	Accession	Size (bp)	Toxin genes	ARGs
pCW3-like plasmids	pCW3	DQ366035.1	47,263	*–*	*tetA*(P) *tetB*(P)
	p21-D-5a	GCA_022808975.1	121,548	*cpe etx aty.cpb2*	–
	pJIR4150	LN835295.1	89,692	*tpeL*	–
	pJFP55F	CP013041.1	72,549	*–*	–
	pCPT84-1	MK285057.1	72,052	*iap ibp*	–
	pJIR3537	CP025504.1	48,779	*–*	*tetA*(P) *tetB*(P)
	pD13122_cpe	MG456815.1	47,758	*cpe aty.cpb2*	–
	pLLY_N11_3	CP023413.1	72,060	*aty.cpb2*	*tetA*(P) *tetB*(P)
	pCBA7123-01	AP017631.1	46,640	*aty.cpb2*	*tetA*(P) *tetB*(P)
	pFORC3	CP009558.1	56,577	*aty.cpb2*	*tetA*(P) *tetB*(P)
	pCPC216_1	CP075974.1	71,757	*cpe*	–
	pJIR3535	CP025502.1	81,826	*netB*	–
	pDel1-1	CP019577.1	82,596	*netB*	–
	pCPPB-1	AB604032.1	67,479	*cpe iap ibp*	–
	pCPF4969	AB236336.1	70,480	*cpe*	–
	pJIR3844	CP025503.1	69,606	*aty.cpb2*	–
	pCpb2-CP1	JQ655732.1	65,875	*aty.cpb2*	*erm*(B)
	pCPAAD1903_1	CP075993.1	49,937	*cpe aty.cpb2*	–
	pCPAAD1527_2	CP076002.1	75,262	*cpe aty.cpb2*	–
	pJFP838D	CP013039.1	48,597	*cpe aty.cpb2*	–
	pCPF5603	AB236337.1	75,268	*cpe aty.cpb2*	–
	pCP8533etx	AB444205.1	64,753	*etx aty.cpb2*	–
*optrA*-positive plasmids	p2C45	NZ_JAAQTM010000004.1	148,618	*–*	*optrA erm*(A) *fexA lnu*(P)
	p21-D-5b	GCA_022808975.1	224,659	*–*	*erm*(Q) *erm*(B) *optrA erm*(A) *fexA lnu*(P) *aac(6′)-aph(2*″)
	pQHY-2	CP118266.1	166,452	*–*	*erm*(Q) *erm*(B) *optrA erm*(A) *fexA aac(6′)-aph(2*″)
pCP13-like plasmids	pCP310-85	CP075969.1	57,459	*cons.cpb2*	–
	pCPT1	MK285059.1	51,991	*cons.cpb2*	–
	pCPCPM77b_1	CP075909.1	53,676	*cons.cpb2*	–
	pCPCPI18-1_2	CP075983.1	53,677	*cons.cpb2*	–
	pCPC269_2	CP075972.1	54,958	*cons.cpb2*	–
	pCPAAD1900a_2	CP075999.1	54,311	*cons.cpb2*	–
	pCP13	AP003515.1	54,310	*cons.cpb2*	–
	pCP-OS1	AP013033.1	54,535	*becA becB*	–
	pCP-TS1	AP013034.1	54,478	*becA becB*	–
	pJUM001-1	AP026871.1	49,289	*–*	–
	pCPAAD1527_1	CP076001.1	58,584	*–*	–
	pCPCPI63K-r5_2	CP075929.1	53,677	*–*	–
	pCP318-46	CP075967.1	56,620	*–*	–
	pCPNY83906550-1	MK285071.1	61,240	*–*	–
	pMFDS1012647.1	CP106927.1	61,476	*–*	–
	pCPCPLi6-1_2	CP075914.1	53,677	*cons.cpb2*	–
	pCPCPLi3-1_2	CP075919.1	58,584	*–*	–
	pCPC746_1	CP075951.1	61,154	*–*	–
	pCP497-86_1	CP075962.1	56,257	*cons.cpb2*	–
	pCPCPI103K-3_2	CP075990.1	58,584	*–*	–
	pCPCPI75-1_2	CP075924.1	62,433	*–*	–
	pCPCPI53k-r1_4	CP075938.1	63,715	*–*	–
	p1	MK275619.1	58,796	*cons.cpb2*	–
	pBCNF5603	AB189671.1	36,695	*–*	–
	pCP1293-86_1	CP076005.1	59,188	*–*	–

^*a*^ –, no characterized toxin genes/ARGs.

### Possible formation mechanism of pQHY-2

**Fig 4:**
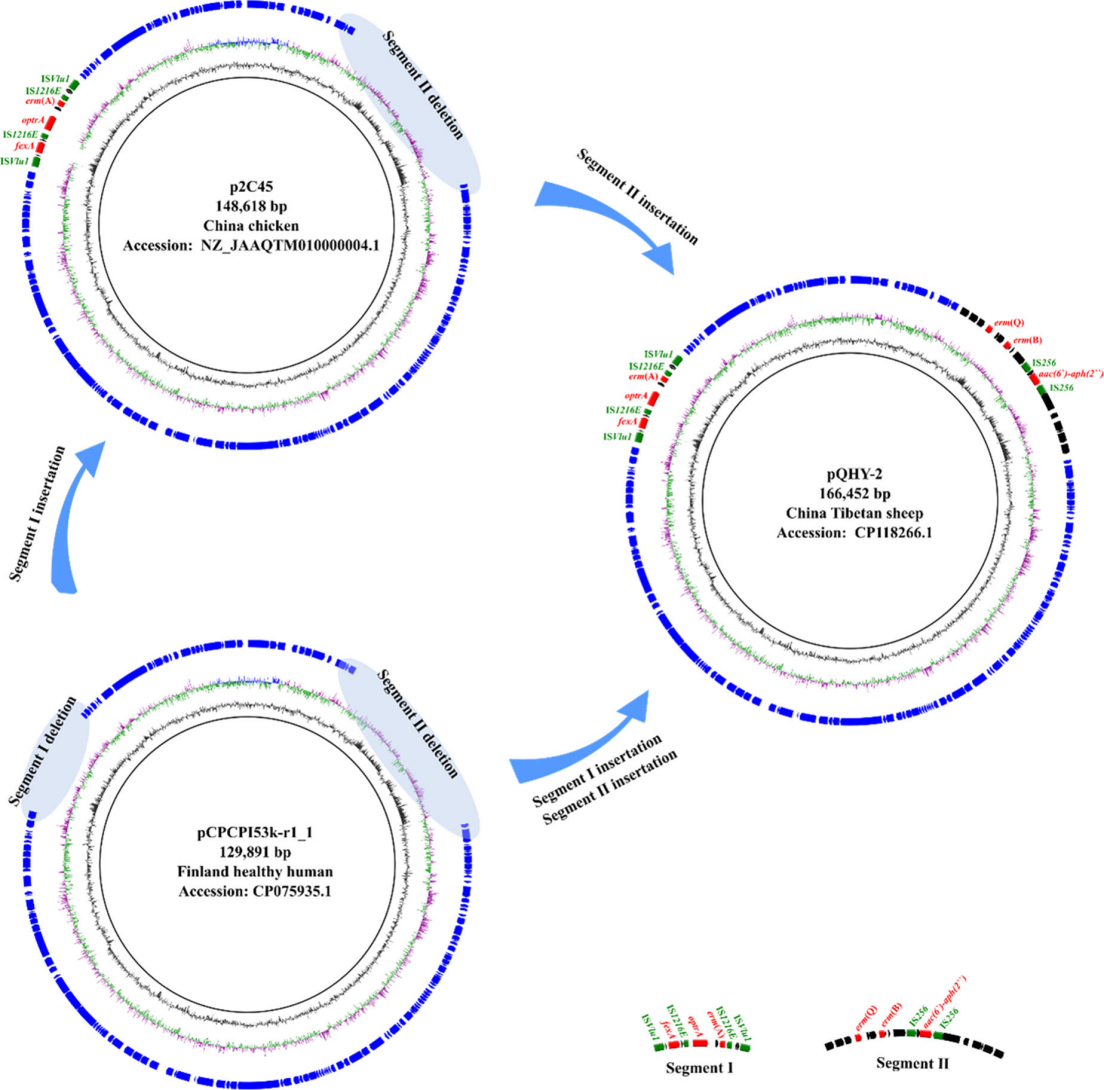
Speculative formation process of plasmid pQHY-2. Segment deletions on the plasmids were represented by blue shadows.

To investigate the possible mechanisms of plasmid pQHY-2, we performed sequence blast on pQHY-2 and the other *C. perfringens* plasmids available in the National Center for Biotechnology Information (NCBI) database and fortunately found an ARGs-negative plasmid named pCPCPI53k-r1_1 (CP075935.1), which was identified in *C. perfringens* strain CPI 53k-r1 isolated from healthy human in Finland (29). Sequence analysis of plasmids pQHY-2 and pCPCPI53k-r1_1 indicated that they shared similar plasmid structure and 78% sequence identity, and both harbored the replication initiator coding genes *repA* and *repB* (Fig. S4), demonstrating pCPCPI53k-r1_1 and the *optrA*-positive plasmids belonged to the same plasmid type. Compared to pQHY-2, there were two segment (segment I and segment II) deletions in pCPCPI53k-r1_1. Segment I (IS*Vlu1-hp-fexA*-IS*1216E-optrA-hp-erm*(A)-IS*1216E-hp-hp*-IS*Vlu1*) encoded *optrA*, *fexA*, and *erm*(A), while segment II (*hp-hp-hp-erm*(Q)-▲Tn*6218-hp*) encoded *erm*(Q), *erm*(B), and *aac(6′)-aph(2*″). Therefore, we speculate that the *optrA* plasmids were formed through inserting segment I and segment II into pCPCPI53k-r1_1, based on the transfer ability of IS elements and Tn*6218* transposon (Fig. 4).

## DISCUSSION

The bacterium *C. perfringens* is of importance to animal and human health, as well as food safety (30). Frequently found in food system environments, *C. perfringens* presents a risk to food animal health and may cross-contaminate associated ingredients or food products, with the potential to cause sporadic outbreaks of disease in human populations, including gastroenteric illness (31). An important pathogenic bacterium, *C. perfringens* possesses several toxin genes and generates a number of dangerous toxins, which lead to huge economic losses in domestic animals and the food industry each year (9, 32). Moreover, *C. perfringens* associated with dairy farm systems show diverse genotypes and contain various virulence makers (16). It therefore remains important to maintain surveillance and toxin genes profiling of *C. perfringens* in farms, foods, and environments, to protect humans and animals from *C. perfringens* infections.

Historically, the use of antibiotics as growth promoters for food producing animals was widespread globally, and high amounts of antimicrobial agents were used for this purpose, in addition to treating bacterial diseases in livestock farming (33). A recognition of the growing problems surrounding AMR and public health has since led to tighter controls around antimicrobial stewardship (34). AMR in bacteria, especially foodborne pathogenic bacteria, still poses a serious threat to public health security. Among the *C. perfringens* strains isolated from humans, food animals, and foods worldwide, strong resistance against sulfonamides has been observed, while moderate resistance against tetracycline, penicillin, lincomycin, and clindamycin was also noted. However, resistance was rarely observed against doxycycline, florfenicol, and linezolid (35 - 39).

In this study, we whole-genome sequenced and analyzed the *optrA*-positive *C. perfringens* strain QHY-2 isolated from Tibetan sheep in Qinghai province. Ten toxin genes were detected in QHY-2, and diverse toxin genes were observed among the *C. perfringens* strains from food animals in China. Among them, *plc*, *cloSI*, and *colA* were detected in all *C. perfringens* isolates, while the other toxin genes, including beta2 toxin coding gene *cons.cpb2*, *C. perfringens* enterotoxin coding gene *cpe*, and epsilon toxin coding gene *etx,* were only detected in some of the *C. perfringens* genomes. The complex virulence makers among *C. perfringens* isolates from food animals indicated standardized feeding management should be strengthened to prevent the transmission and pathogenicity of them. Moreover, the toxin gene repertories of *C. perfringens* varied with the animal species source, suggesting targeted preventive measures should be undertaken to limit *C. perfringens*-associated diseases. Besides the toxin genes, various ARGs were also observed among the *C. perfringens* strains. Among them, *tetA*(P) was found to have the highest prevalence rate (94.5%, 86/91), followed by *tetB*(P) (64.8%, 59/91), *tet* (22) (37.4%, 34/91), *ant (6)-Ib* (35.2%, 32/91), *erm*(Q) (35.2%, 32/91), and *lnu*(P) (29.7%, 27/91) (Fig. S5). The high prevalence of tetracycline-resistant and lincomycin-resistant genes partly explained the mild resistance against tetracycline, lincomycin, and clindamycin. However, no sulfonamides-resistant genes were identified among the *C. perfringens* isolates. Further studies should be carried out to explain the strong resistance against sulfonamides in *C. perfringens*.

OptrA protein encoded by *optrA* gene can mediate resistance against oxazolidinones (linezolid and tedizolid), the last-resort antimicrobial agents used for the treatment of severe MDR Gram-positive bacterial infections (40). After the discovery of *optrA* gene in *Enterococcus faecalis* strain E349 in 2015, it has been detected among the Gram-positive *Enterococcus* and *Staphylococcus* isolates (41 - 43), and the Gram-negative *Campylobacter* isolates from humans, hospital environments, foods, and animals in China (22, 44). Prevalence of *optrA* may pose challenge to the treatment of bacterial infectious diseases and potential co-selection of other ARGs, which can cause huge threats to public health and safety. In this study, we found the *optrA* genes in *C. perfringens* shared similar gene environments with the *optrA* genes in *Enterococcus*, *Staphylococcus*, and *Campylobacter* strains, indicating the spread of *optrA* gene among bacterial strains. Additionally, the presence of other plasmid-borne resistance genes *fexA*, *erm*(Q), *aac(6′)-aph(2*″), *erm*(B), and *lnu*(P), particularly *erm*(A) and *fexA* that are located on a segment flanked by IS element IS*Vlu1* or IS*1216E* together with *optrA*, may contribute to the co-selection of *optrA. C. perfringens* is one of the main causes of gas gangrene and foodborne diseases in humans (45), which can transfer between food animals and humans through animal products. AMR of *C. perfringens* from food animals can pose serious threats to food safety and public health. The various ARGs in *C. perfringens* strains from food animals in China suggested that the rational use of antibiotics should be strengthened to prevent the increase and spread of AMR.

Many important toxin genes and ARGs that are crucial to the pathology of *C. perfringens* are located on plasmids, and so these genetic elements play important roles in the cases of *C. perfringens* infection. According to the type of replication initiator, the plasmids in *C. perfringens* can be categorized into pCW3-like plasmids, pCP13-like plasmids, and pIP404-like plasmids (21). In this study, we identified one *optrA*-positive plasmid pQHY-2 in *C. perfringens* strain QHY-2 from Tibetan sheep in Qinghai province and further compared it with the other two *optrA*-positive plasmids p2C45 and p21-D-5 previously identified in *C. perfringens* strains 2C45 (BioSample: SAMN18155071) and 21-D-5 (BioSample: SAMN28005067) isolated from chicken in Shanxi province and goat in Shaanxi province, respectively. Molecular analysis indicated that the *optrA*-positive plasmids of *C. perfringens* belonged to a plasmid type, with different replication initiator coding genes, larger plasmid size, and more ARGs that differed from pCW3-like plasmids, pCP13-like plasmids, or pIP404-like plasmids. Further sequence and structure analysis revealed that the *optrA*-positive plasmids might be formed by inserting segments into the ARGs-negative plasmid pCPCPI53k-r1_1, which belonged to the same plasmid type with the three *optrA*-positive plasmids. Additionally, pCPCPI53k-r1_1 exists in the *C. perfringens* strain CPI 53k-r1, together with two pCW3-like plasmids (pCPCPI53k-r1_2 and pCPCPI53k-r1_3), one pCP13-like plasmid pCPCPI53k-r1_4, and two small plasmids (pCPCPI53k-r1_5 and pCPCPI53k-r1_6) harboring bacteriocin coding gene *bcn* (Fig. S6), demonstrating the possible coexistence of the *optrA*-positive plasmids and the pCW3-like plasmids and pCP13-like plasmids in *C. perfringens* strains.

Although the *optrA* plasmids have not been successfully transferred to date, the dissemination of similar homologous plasmids across multiple strains from humans and animals in different regions suggests a possible horizontal transfer of the ARGs among *C. perfringens* strains, especially those harbor other toxin plasmids, which poses a potential threat to food safety and public health. In conclusion, this study indicates that *C. perfringens* isolates from food animals in China harbored various virulence makers and ARGs and supports the need for effective regulations and measures to control the dissemination of antibiotic resistance of *C. perfringens*, especially those strains encoding key genetic markers linked to severe disease.

## MATERIALS AND METHODS

### Source of isolate and detection of florfenicol-resistant genes

Eighty fecal samples were collected from Tibetan sheep grazed in Qinghai province, China, in 2020. Thirty-six strains of *C. perfringens* were eventually recovered from the samples after isolation and molecular-based identification, and antibiotic sensitivity testing was then performed on the isolates. Interestingly, we found one *C. perfringens* designated as QHY-2 showed strong resistance against florfenicol (MIC = 32 µg/mL) (46), which was rarely observed in *C. perfringens*. To investigate the causes of strong resistance against florfenicol in QHY-2, in this study, we screened for the florfenicol-resistant genes *floR*, *fexA*, *fexB*, *cfr*, and *optrA* with PCR assays, as described in the previous reports (47 - 49).

### Whole-genome sequencing and analysis

Genome DNA of QHY-2 was extracted using TIANamp Bacteria DNA Kit (Tiangen, Beijing, China). WGS of the strain QHY-2 was performed with the Nanopore PromethION platform (Biomarker Technologies, Beijing, China). The sequences were assembled with Canu v1.5. Pilon v1.22 was used to improve the draft genome assemblies by correcting bases. The WGS annotations were designed with the Rapid Annotations using Subsystem Technology annotation pipeline (version 2.0) (http://rast.nmpdr.org/) and Prokka (50). AMR and toxin genes were identified by Abricate and standalone BLAST analysis. Conjugative locus of the plasmids was identified by local blast, Tcp locus, and Pcp locus coding genes were used as the BLAST database (18, 21). Nucleotide sequence visualization and comparison were realized through BRIG and Easyfig. A total of 91 genomes of *C. perfringens* (Table S1), including QHY-2 and the other 90 *C. perfringens* isolates recovered from food animals in China were used to construct a phylogenetic tree based on single nucleotide polymorphisms, using REALPHY (https://realphy.unibas.ch/realphy/). A phylogenetic tree was visualized using the online tool iTOL (https://itol.embl.de/).

### Transformation experiments

The *optrA*-positive plasmids were extracted and used to electrotransform into the recipient strain *C. perfringens* ATCC 13124. Three randomly selected *C. perfringens* isolates with low MICs (<8 µg/mL) to florfenicol were chosen as recipients for conjugation (25).

## Data Availability

*Clostridium perfringens* strain QHY-2 chromosome and plasmid pQHY-2 were deposited in GenBank under the GenBank accession numbers CP118265.1 and CP118266.1, respectively.

## SUPPLEMENTAL MATERIAL

Fig. S1 msystems.00535-23-s0001.docx Cluster heat map based on the toxin genes profiles.

Fig. S2 msystems.00535-23-s0002.docx Distribution of toxin genes among the ninety-one *C. perfringens* strains.

Fig. S3 msystems.00535-23-s0003.docx Detections of replication initiator coding genes.

Fig. S4 msystems.00535-23-s0004.docx Circler comparison of plasmids pQHY-2 and pCPCPI53k-r1_1.

Fig. S5 msystems.00535-23-s0005.docx Prevalence of antibiotic resistance genes in the *C. perfringens* strains.

Fig. S6 msystems.00535-23-s0006.docx Summary of the plasmids in the human*C. perfringens* strain CPI 53k-r1.

Table S1 msystems.00535-23-s0007.docx Basic information of the ninety-one *C. perfringens* genomes used in this study.

ASM does not own the copyrights to Supplemental Material that may be linked to, or accessed through, an article. The authors have granted ASM a non-exclusive, world-wide license to publish the Supplemental Material files. Please contact the corresponding author directly for reuse.
