# Supplementary material for: Genome characteristics of the optrA-positive Clostridium perfringens strain QHY-2 carrying a novel plasmid type
Source: mSystems. 2023 Jul 17;8(4):e00535-23. doi: 10.1128/msystems.00535-23 (PMC10469678; doi:10.1128/msystems.00535-23)
Supplement: Fig. S1 — Cluster heat map based on the toxin genes profiles. [file msystems.00535-23-s0001.docx]

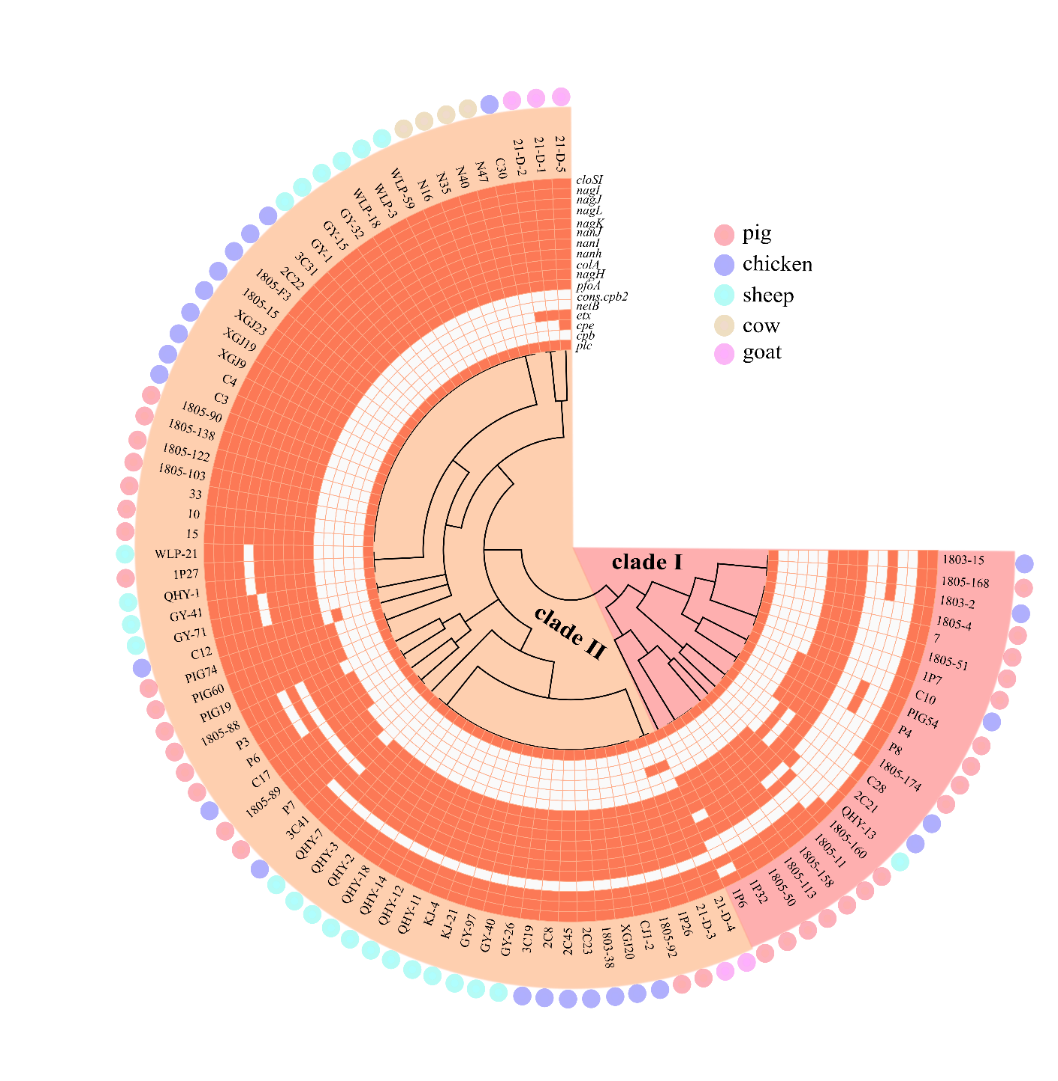


**Figure S1.** Cluster heat map based on the toxin genes profiles of the selected *C. perfringens* genomes. The *C. perfringens* strains are divided into clade Ⅰ that mainly consisted of *C. perfringens* isolates from pigs and clade Ⅱ consisted of *C. perfringens* isolates from various animals.
