# Supplementary material for: Genome characteristics of the optrA-positive Clostridium perfringens strain QHY-2 carrying a novel plasmid type
Source: mSystems. 2023 Jul 17;8(4):e00535-23. doi: 10.1128/msystems.00535-23 (PMC10469678; doi:10.1128/msystems.00535-23)
Supplement: Fig. S2 — Distribution of toxin genes among the ninety-one C. perfringens strains. [file msystems.00535-23-s0002.docx]

**
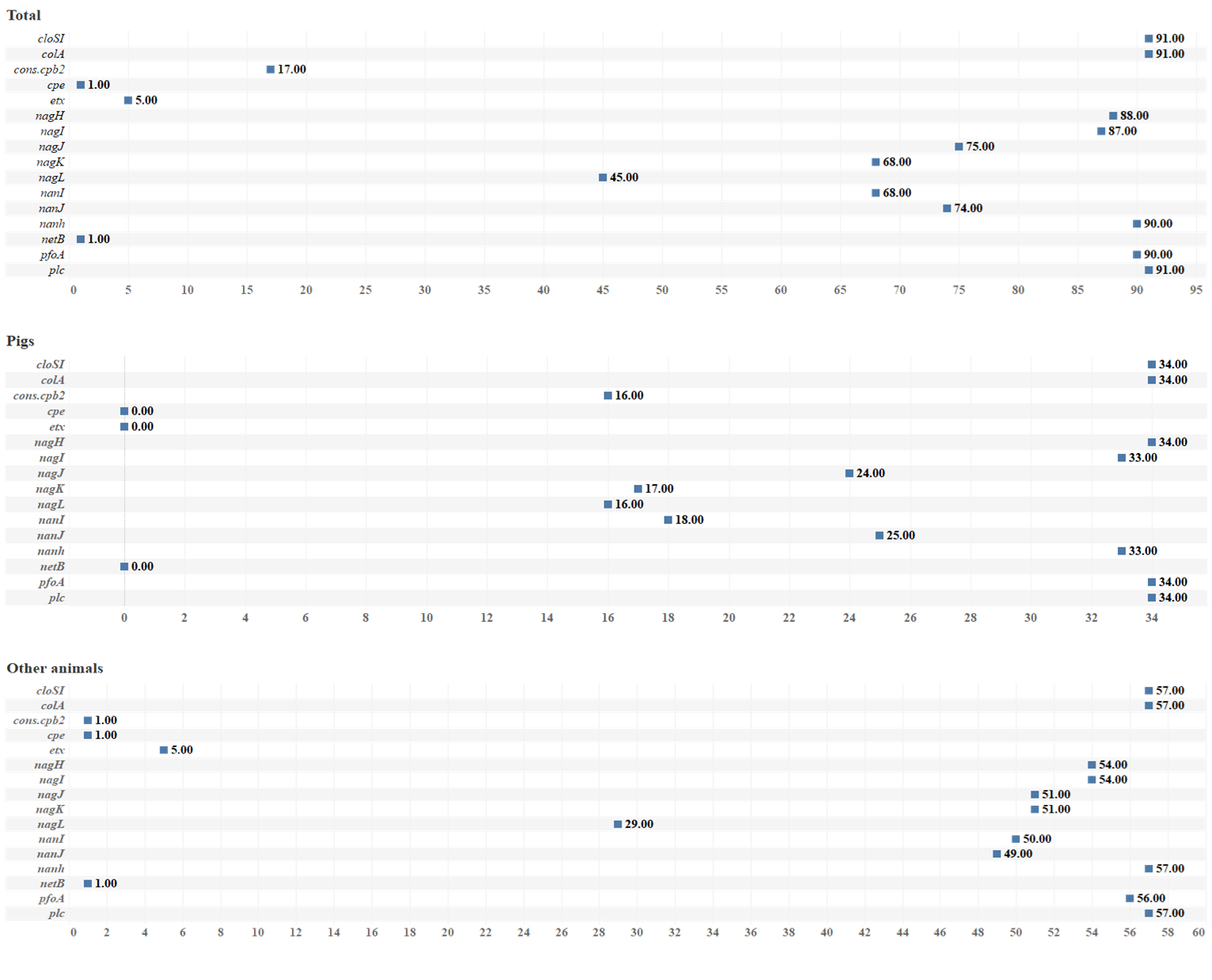
Figure S2.** Distribution of toxin genes among the ninety-one *C. perfringens* strains recovered from food animals in China. From top to bottom, there are all selected strains (n= 91), pig strains (n= 34) and other animal strains (n= 57), respectively.
