# Supplementary material for: Genome characteristics of the optrA-positive Clostridium perfringens strain QHY-2 carrying a novel plasmid type
Source: mSystems. 2023 Jul 17;8(4):e00535-23. doi: 10.1128/msystems.00535-23 (PMC10469678; doi:10.1128/msystems.00535-23)
Supplement: Fig. S3 — Detections of replication initiator coding genes. [file msystems.00535-23-s0003.docx]

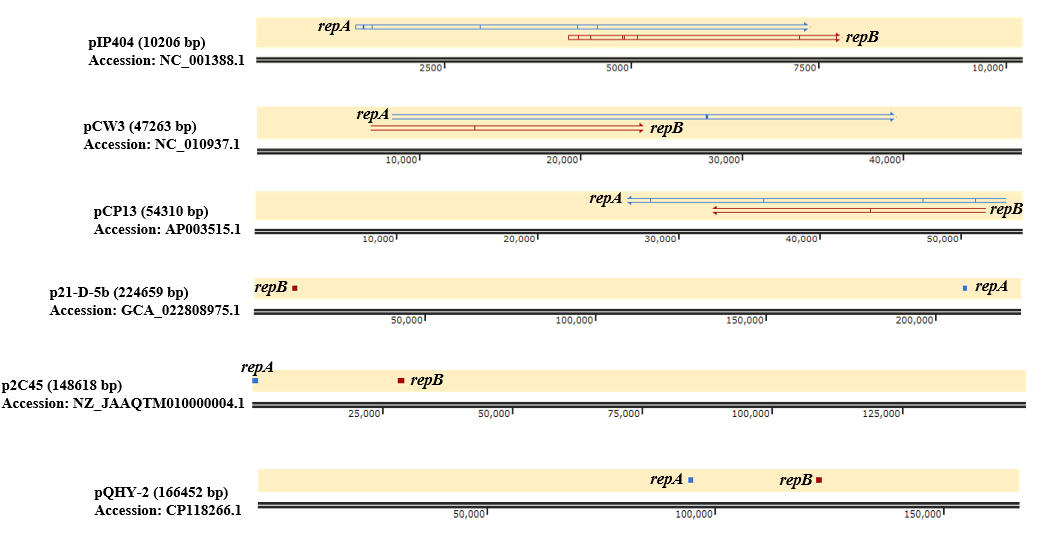
**Figure S3.** Detections of the *optrA*-positive plasmids` replication initiator coding genes *repA* and *repB* on plasmids pCW3, pCP13, pIP404, and the three *optrA*-positive plasmids.
