# Supplementary figures and images for: Genome characteristics of the optrA-positive Clostridium perfringens strain QHY-2 carrying a novel plasmid type
Source: mSystems. 2023 Jul 17;8(4):e00535-23. doi: 10.1128/msystems.00535-23 (PMC10469678; doi:10.1128/msystems.00535-23)

**
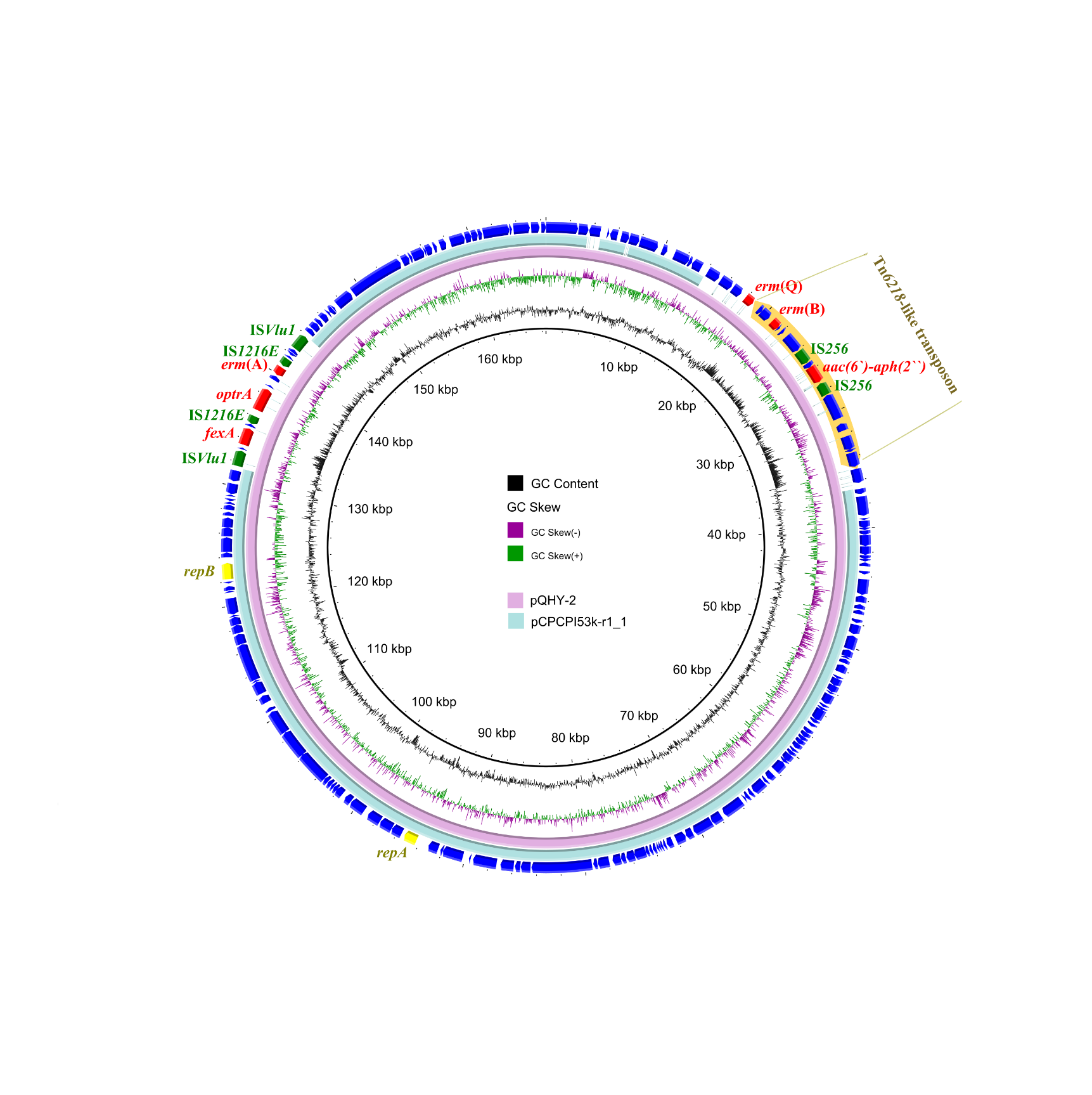
Figure S4.** Circler comparison of plasmids pQHY-2 and pCPCPI53k-r1_1, using pQHY-2 as reference sequence.

Supplement: Fig. S4 — Circler comparison of plasmids pQHY-2 and pCPCPI53k-r1_1. [file msystems.00535-23-s0004.docx]

**
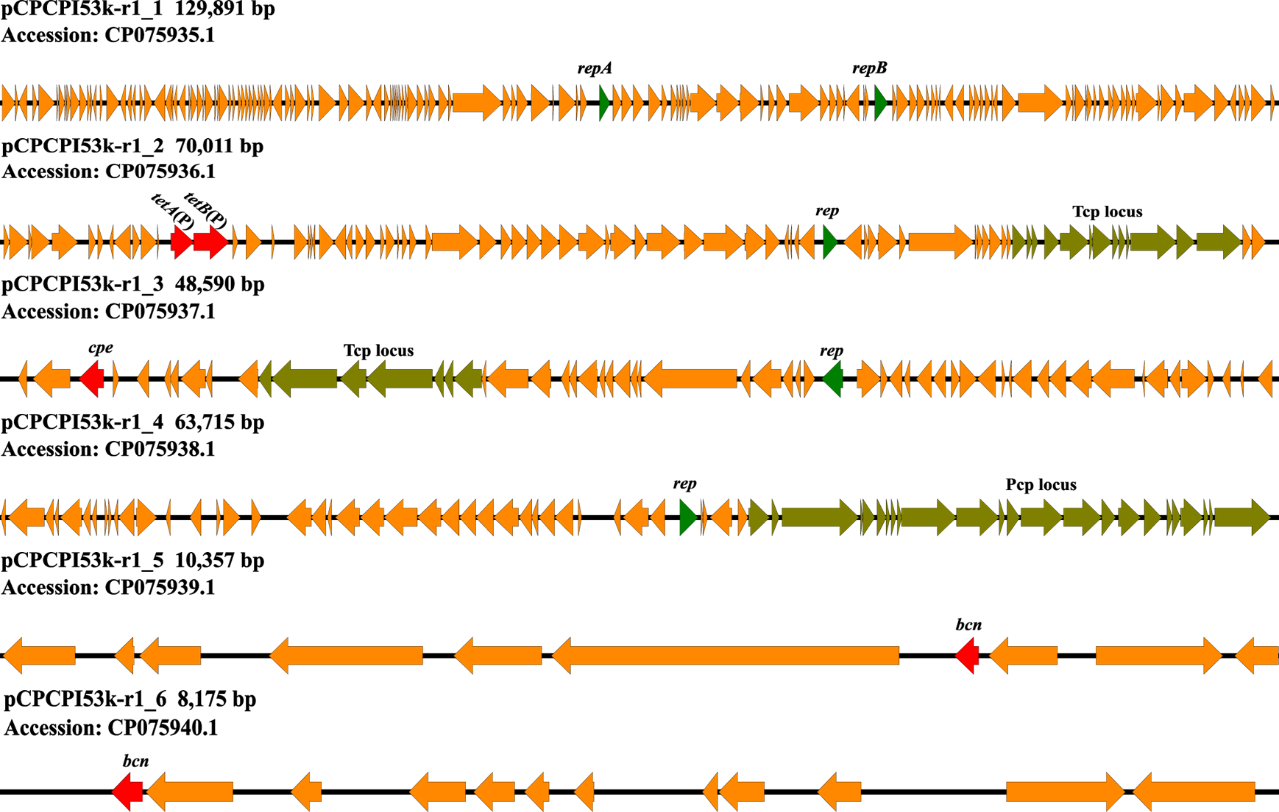
Figure S6.** Summary of the plasmids in the human *C. perfringens* strain CPI 53k-r1.

Supplement: Fig. S6 — Summary of the plasmids in the humanC. perfringens strain CPI 53k-r1. [file msystems.00535-23-s0006.docx]
