# Supplementary material for: Genome characteristics of the optrA-positive Clostridium perfringens strain QHY-2 carrying a novel plasmid type
Source: mSystems. 2023 Jul 17;8(4):e00535-23. doi: 10.1128/msystems.00535-23 (PMC10469678; doi:10.1128/msystems.00535-23)
Supplement: Fig. S5 — Prevalence of antibiotic resistance genes in the C. perfringens strains. [file msystems.00535-23-s0005.docx]

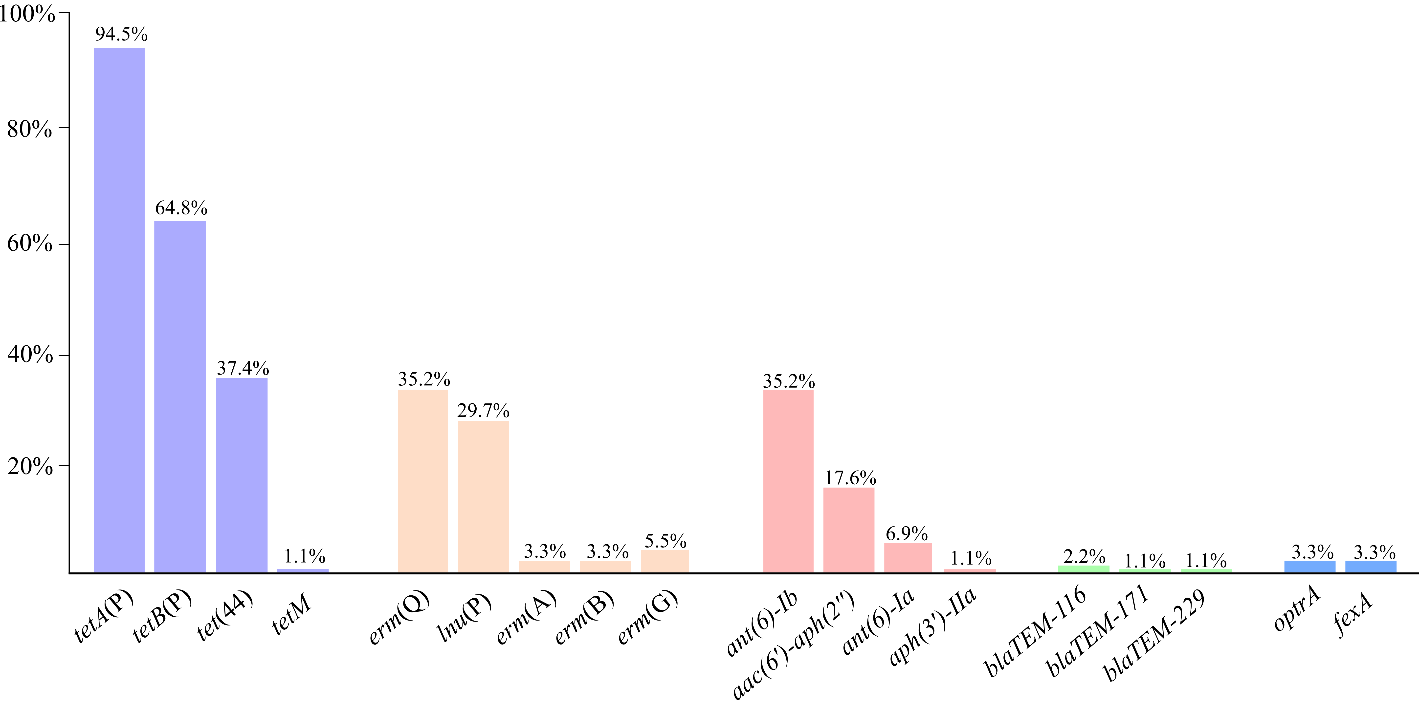
**Figure S5.** Prevalence of antibiotic resistance genes in the *C. perfringens* strains from food animals in China.
