# Supplementary material for: Genome characteristics of the optrA-positive Clostridium perfringens strain QHY-2 carrying a novel plasmid type
Source: mSystems. 2023 Jul 17;8(4):e00535-23. doi: 10.1128/msystems.00535-23 (PMC10469678; doi:10.1128/msystems.00535-23)
Supplement: Table S1 — Basic information of the ninety-one C. perfringens genomes used in this study. [file msystems.00535-23-s0007.docx]

**TABLE S1** Basic information of the ninety-one *C. perfringens* genomes used in this study

| Name | Animal | Region | Antibitic resistant genes | | | | | | | | | | | | | | | | | | | Tonxin genes | | | | | | | | | | | | | | | | |
| --- | --- | --- | --- | --- | --- | --- | --- | --- | --- | --- | --- | --- | --- | --- | --- | --- | --- | --- | --- | --- | --- | --- | --- | --- | --- | --- | --- | --- | --- | --- | --- | --- | --- | --- | --- | --- | --- | --- |
|  |  |  | *erm*(Q) | *erm*(G) | *erm*(B) | *erm*(A) | *lnu*(P) | *optrA* | *fexA* | *aac(6')-aph(2'')* | *ant(6)-Ia* | *ant(6)-Ib* | *aph(3')-IIa* | *blaTEM-116* | *blaTEM-171* | *blaTEM-229* | *cfr(C)* | *tetB*(P) | *tetA*(P) | *tetM* | *tet*(44) | *plc* | *cpb* | *cpe* | *etx* | *netB* | *cons.cpb2* | *pfoA* | *nagH* | *colA* | *nanh* | *nanI* | *nanJ* | *nagK* | *nagL* | *nagJ* | *nagI* | *cloSI* |
| 7 | pig | Beijing | + | + | - | - | + | - | - | - | + | + | - | - | - | - | - | + | + | - | + | + | - | - | - | - | - | + | + | + | + | - | - | - | - | - | + | + |
| 10 | pig | Beijing | + | - | - | - | + | - | - | - | - | + | - | - | - | - | - | - | + | - | + | + | - | - | - | - | - | + | + | + | + | + | + | + | + | + | + | + |
| 15 | pig | Beijing | + | - | - | - | - | - | - | - | - | - | - | - | - | - | - | + | + | - | - | + | - | - | - | - | - | + | + | + | + | + | + | + | + | + | + | + |
| 33 | pig | Beijing | + | - | - | - | - | - | - | - | - | - | - | - | - | - | - | + | + | - | - | + | - | - | - | - | - | + | + | + | + | + | + | + | + | + | + | + |
| 1805-103 | pig | Beijing | - | - | - | - | - | - | - | - | - | + | - | - | - | - | - | + | + | - | + | + | - | - | - | - | - | + | + | + | + | + | + | + | + | + | + | + |
| 1805-11 | pig | Beijing | + | - | - | - | + | - | - | - | - | - | - | - | - | - | - | + | + | - | - | + | - | - | - | - | + | + | + | + | + | - | + | - | - | + | + | + |
| 1805-113 | pig | Beijing | - | - | - | - | + | - | - | + | - | + | - | - | - | - | - | - | + | - | + | + | - | - | - | - | + | + | + | + | + | + | + | - | - | + | + | + |
| 1805-122 | pig | Beijing | - | - | - | - | - | - | - | - | - | + | - | - | - | - | - | + | + | - | + | + | - | - | - | - | - | + | + | + | + | + | + | + | + | + | + | + |
| 1805-138 | pig | Beijing | - | - | - | - | - | - | - | - | - | + | - | - | - | - | - | + | + | - | + | + | - | - | - | - | - | + | + | + | + | + | + | + | + | + | + | + |
| 1805-158 | pig | Beijing | + | - | - | - | - | - | - | - | - | + | - | - | - | - | - | - | + | - | + | + | - | - | - | - | + | + | + | + | + | + | + | - | - | + | + | + |
| 1805-160 | pig | Beijing | - | + | - | - | + | - | - | - | + | + | - | - | - | - | - | - | + | - | + | + | - | - | - | - | + | + | + | + | + | - | + | - | - | - | + | + |
| 1805-168 | pig | Beijing | - | - | - | - | - | - | - | - | - | + | - | - | - | - | - | - | + | - | + | + | - | - | - | - | - | + | + | + | + | - | - | + | - | - | + | + |
| 1805-174 | pig | Beijing | + | + | - | - | + | - | - | - | + | + | - | - | - | - | - | - | + | - | + | + | - | - | - | - | + | + | + | + | + | - | - | - | - | - | + | + |
| 1805-4 | pig | Beijing | - | - | - | - | + | - | - | - | - | + | - | - | - | - | - | - | + | - | + | + | - | - | - | - | - | + | + | + | + | - | - | - | - | - | + | + |
| 1805-50 | pig | Beijing | - | - | - | - | + | - | - | + | - | + | - | - | - | - | - | - | + | - | + | + | - | - | - | - | + | + | + | + | + | + | + | - | - | + | + | + |
| 1805-51 | pig | Beijing | - | - | - | - | + | - | - | - | - | + | - | - | - | - | - | - | - | - | + | + | - | - | - | - | - | + | + | + | + | - | - | - | - | - | + | + |
| 1805-88 | pig | Beijing | + | - | - | - | + | - | - | - | - | - | - | - | - | - | - | - | + | - | + | + | - | - | - | - | - | + | + | + | + | - | + | - | + | + | + | + |
| 1805-89 | pig | Beijing | + | - | - | - | + | - | - | - | - | - | + | + | + | + | - | + | + | - | + | + | - | - | - | - | - | + | + | + | + | - | + | + | + | + | + | + |
| 1805-90 | pig | Beijing | + | - | - | - | - | - | - | - | - | - | - | - | - | - | - | + | + | - | - | + | - | - | - | - | - | + | + | + | + | + | + | + | + | + | + | + |
| 1805-92 | pig | Beijing | - | - | - | - | - | - | - | - | - | - | - | - | - | - | - | + | + | - | - | + | - | - | - | - | - | + | + | + | + | + | + | + | - | + | + | + |
| 1P26 | pig | Shanxi | + | - | - | - | - | - | - | - | - | - | - | - | - | - | - | + | + | - | - | + | - | - | - | - | - | + | + | + | + | + | + | + | - | + | + | + |
| 1P27 | pig | Shanxi | - | - | + | - | + | - | - | + | - | - | - | - | - | - | - | + | + | - | - | + | - | - | - | - | - | + | + | + | + | + | + | - | + | + | + | + |
| 1P32 | pig | Shanxi | - | - | - | - | - | - | - | + | - | + | - | - | - | - | - | - | + | - | + | + | - | - | - | - | + | + | + | + | + | + | + | - | - | + | + | + |
| 1P6 | pig | Shanxi | - | - | - | - | - | - | - | + | + | - | - | - | - | - | - | - | + | - | + | + | - | - | - | - | + | + | + | + | - | + | + | - | - | + | - | + |
| 1P7 | pig | Shanxi | - | - | - | - | - | - | - | - | - | + | - | - | - | - | - | - | + | - | + | + | - | - | - | - | - | + | + | + | + | - | - | - | - | - | + | + |
| P3 | pig | Shanxi | + | - | - | - | - | - | - | - | - | + | - | - | - | - | - | - | + | - | + | + | - | - | - | - | + | + | + | + | + | - | + | - | + | + | + | + |
| P4 | pig | Shanxi | - | - | - | - | + | - | - | - | - | + | - | - | - | - | - | - | + | - | + | + | - | - | - | - | + | + | + | + | + | - | - | + | - | - | + | + |
| P6 | pig | Shanxi | + | - | - | - | - | - | - | - | - | + | - | - | - | - | - | - | + | - | + | + | - | - | - | - | + | + | + | + | + | - | + | - | + | + | + | + |
| P7 | pig | Shanxi | - | - | - | - | + | - | - | - | - | + | - | - | - | - | - | - | + | - | + | + | - | - | - | - | - | + | + | + | + | - | + | + | + | + | + | + |
| P8 | pig | Shanxi | - | - | - | - | + | - | - | - | - | + | - | - | - | - | - | + | + | - | + | + | - | - | - | - | + | + | + | + | + | - | - | - | - | - | + | + |
| PIG54 | pig | Sichuan | - | - | - | - | + | - | - | - | - | + | - | - | - | - | - | + | + | - | + | + | - | - | - | - | + | + | + | + | + | - | - | + | - | - | + | + |
| PIG60 | pig | Sichuan | - | - | - | - | - | - | - | - | - | + | - | - | - | - | - | - | + | - | + | + | - | - | - | - | + | + | + | + | + | + | + | + | + | + | + | + |
| PIG19 | pig | Sichuan | - | - | - | - | - | - | - | - | - | + | - | - | - | - | - | - | + | - | + | + | - | - | - | - | + | + | + | + | + | + | + | + | + | + | + | + |
| PIG74 | pig | Sichuan | - | - | - | - | - | - | - | - | - | + | - | - | - | - | - | - | + | - | + | + | - | - | - | - | + | + | + | + | + | + | + | + | + | + | + | + |
| C12 | chicken | Shaanxi | - | - | - | - | - | - | - | - | - | - | - | - | - | - | - | + | + | - | - | + | - | - | - | + | - | + | + | + | + | + | + | + | + | + | + | + |
| CJ1-2 | chicken | Shaanxi | - | - | - | - | - | - | - | - | - | - | - | - | - | - | - | + | + | - | - | + | - | - | - | - | - | + | + | + | + | + | + | + | - | + | + | + |
| C3 | chicken | Shaanxi | - | - | - | - | - | - | - | - | - | - | - | - | - | - | - | + | + | - | - | + | - | - | - | - | - | + | + | + | + | + | + | + | + | + | + | + |
| C4 | chicken | Shaanxi | + | - | - | - | - | - | - | - | - | - | - | - | - | - | - | + | + | - | - | + | - | - | - | - | - | + | + | + | + | + | + | + | + | + | + | + |
| XGJ9 | chicken | Gansu | - | - | - | - | - | - | - | - | - | - | - | - | - | - | - | + | + | - | - | + | - | - | - | - | - | + | + | + | + | + | + | + | + | + | + | + |
| XGJ19 | chicken | Gansu | - | - | - | - | - | - | - | - | - | - | - | - | - | - | - | + | + | - | - | + | - | - | - | - | - | + | + | + | + | + | + | + | + | + | + | + |
| XGJ23 | chicken | Gansu | - | - | - | - | - | - | - | - | - | - | - | - | - | - | - | + | + | - | - | + | - | - | - | - | - | + | + | + | + | + | + | + | + | + | + | + |
| XGJ20 | chicken | Gansu | + | - | - | - | - | - | - | - | - | - | - | - | - | - | - | + | + | - | - | + | - | - | - | - | - | + | + | + | + | + | + | + | - | + | + | + |
| 1803-15 | chicken | Beijing | - | - | - | - | - | - | - | - | - | + | - | - | - | - | - | + | + | - | + | + | - | - | - | - | - | + | + | + | + | - | - | + | - | - | + | + |
| 1803-2 | chicken | Beijing | - | - | - | - | - | - | - | - | - | + | - | - | - | - | - | + | + | - | + | + | - | - | - | - | - | + | + | + | + | - | - | + | - | - | + | + |
| 1803-38 | chicken | Beijing | + | - | - | - | - | - | - | - | - | - | - | - | - | - | - | - | + | + | - | + | - | - | - | - | - | + | + | + | + | + | + | + | - | + | + | + |
| 1805-15 | chicken | Beijing | - | - | - | - | - | - | - | - | - | - | - | - | - | - | - | + | + | - | - | + | - | - | - | - | - | + | + | + | + | + | + | + | + | + | + | + |
| 1805-F3 | chicken | Beijing | + | - | - | - | - | - | - | - | - | - | - | - | - | - | - | + | + | - | - | + | - | - | - | - | - | + | + | + | + | + | + | + | + | + | + | + |
| 2C21 | chicken | Shanxi | - | - | - | - | - | - | - | - | - | - | - | - | - | - | - | + | + | - | - | + | - | - | - | - | - | + | - | + | + | - | - | - | - | - | - | + |
| 2C22 | chicken | Shanxi | - | - | - | - | - | - | - | - | - | - | - | - | - | - | - | - | + | - | - | + | - | - | - | - | - | + | + | + | + | + | + | + | + | + | + | + |
| 2C23 | chicken | Shanxi | - | - | - | - | - | - | - | - | - | - | - | - | - | - | - | + | + | - | - | + | - | - | - | - | - | + | + | + | + | + | + | + | - | + | + | + |
| 2C45 | chicken | Shanxi | + | - | - | + | + | + | + | + | - | - | - | - | - | - | - | + | + | - | - | + | - | - | - | - | - | + | + | + | + | + | + | + | - | + | + | + |
| 2C8 | chicken | Shanxi | + | - | - | - | - | - | - | - | - | - | - | + | - | - | - | + | + | - | - | + | - | - | - | - | - | + | + | + | + | + | + | + | - | + | + | + |
| 3C19 | chicken | Shanxi | + | - | - | - | + | - | - | + | - | - | - | - | - | - | - | + | + | - | - | + | - | - | - | - | - | + | + | + | + | + | + | + | - | + | + | + |
| 3C31 | chicken | Shanxi | + | - | - | - | + | - | - | - | - | - | - | - | - | - | - | + | + | - | - | + | - | - | - | - | - | + | + | + | + | + | + | + | + | + | + | + |
| 3C41 | chicken | Shanxi | + | - | - | - | + | - | - | + | - | - | - | - | - | - | - | + | + | - | - | + | - | - | - | - | - | - | + | + | + | - | + | + | - | + | + | + |
| GY-1 | sheep | Gansu | + | - | - | - | - | - | - | - | - | - | - | - | - | - | - | + | + | - | - | + | - | - | - | - | - | + | + | + | + | + | + | + | + | + | + | + |
| GY-15 | sheep | Gansu | - | - | - | - | - | - | - | - | - | - | - | - | - | - | - | - | + | - | - | + | - | - | - | - | - | + | + | + | + | + | + | + | + | + | + | + |
| GY-26 | sheep | Gansu | - | - | - | - | - | - | - | - | - | - | - | - | - | - | - | + | + | - | - | + | - | - | - | - | - | + | + | + | + | + | + | + | - | + | + | + |
| GY-32 | sheep | Gansu | + | - | - | - | - | - | - | - | - | - | - | - | - | - | - | + | + | - | - | + | - | - | - | - | - | + | + | + | + | + | + | + | + | + | + | + |
| GY-40 | sheep | Gansu | - | - | - | - | + | - | - | - | - | - | - | - | - | - | - | + | + | - | - | + | - | - | - | - | - | + | + | + | + | + | + | + | - | + | + | + |
| GY-41 | sheep | Gansu | - | - | - | - | - | - | - | - | - | - | - | - | - | - | - | + | - | - | - | + | - | - | - | - | - | + | + | + | + | + | - | + | + | + | + | + |
| GY-71 | sheep | Gansu | - | - | - | - | - | - | - | - | - | - | - | - | - | - | - | + | + | - | - | + | - | - | - | - | - | + | + | + | + | + | - | + | + | + | + | + |
| GY-97 | sheep | Gansu | + | - | - | - | - | - | - | - | - | - | - | - | - | - | - | + | + | - | - | + | - | - | - | - | - | + | + | + | + | + | + | + | - | + | + | + |
| KJ-21 | sheep | Gansu | - | - | - | - | - | - | - | - | - | - | - | - | - | - | - | + | + | - | - | + | - | - | - | - | - | + | + | + | + | + | + | + | - | + | + | + |
| KJ-4 | sheep | Gansu | - | - | - | - | - | - | - | - | - | - | - | - | - | - | - | + | + | - | - | + | - | - | - | - | - | + | + | + | + | + | + | + | - | + | + | + |
| QHY-1 | TS | Qinghai | - | - | - | - | + | - | - | + | - | - | - | - | - | - | - | + | + | - | - | + | - | - | - | - | - | + | + | + | + | + | + | - | + | + | + | + |
| QHY-11 | TS | Qinghai | - | - | - | - | + | - | - | + | - | + | - | - | - | - | - | - | + | - | + | + | - | - | - | - | - | + | + | + | + | + | + | + | - | + | + | + |
| QHY-12 | TS | Qinghai | - | - | - | - | + | - | - | + | - | + | - | - | - | - | - | - | + | - | + | + | - | - | - | - | - | + | + | + | + | + | + | + | - | + | + | + |
| QHY-13 | TS | Qinghai | - | - | - | - | - | - | - | - | - | - | - | - | - | - | - | - | - | - | - | + | - | - | - | - | - | + | - | + | + | + | - | - | - | - | - | + |
| QHY-14 | TS | Qinghai | - | - | - | - | - | - | - | - | - | - | - | - | - | - | - | + | + | - | - | + | - | - | - | - | - | + | + | + | + | + | + | + | - | + | + | + |
| QHY-18 | TS | Qinghai | - | - | - | - | + | - | - | - | - | - | - | - | - | - | - | + | + | - | - | + | - | - | - | - | - | + | + | + | + | + | + | + | - | + | + | + |
| QHY-2 | TS | Qinghai | + | - | + | + | - | + | + | + | - | + | - | - | - | - | - | - | + | - | + | + | - | - | - | - | - | + | + | + | + | + | + | + | - | + | + | + |
| QHY-3 | TS | Qinghai | - | - | - | - | + | - | - | + | - | + | - | - | - | - | - | - | + | - | + | + | - | - | - | - | - | + | + | + | + | + | + | + | - | + | + | + |
| QHY-7 | TS | Qinghai | - | - | - | - | - | - | - | - | - | - | - | - | - | - | - | + | + | - | - | + | - | - | - | - | - | + | + | + | + | + | + | + | - | + | + | + |
| WLP-18 | sheep | Gansu | - | - | - | - | - | - | - | - | - | - | - | - | - | - | - | - | - | - | - | + | - | - | - | - | - | + | + | + | + | + | + | + | + | + | + | + |
| WLP-21 | sheep | Gansu | - | - | - | - | - | - | - | - | - | - | - | - | - | - | - | + | + | - | - | + | - | - | - | - | - | + | + | + | + | + | + | - | + | + | + | + |
| WLP-3 | sheep | Gansu | + | - | - | - | - | - | - | + | - | - | - | - | - | - | - | + | + | - | - | + | - | - | - | - | - | + | + | + | + | + | + | + | + | + | + | + |
| WLP-59 | sheep | Gansu | - | - | - | - | - | - | - | - | - | - | - | - | - | - | - | - | - | - | - | + | - | - | - | - | - | + | + | + | + | + | + | + | + | + | + | + |
| 21-D-1 | goat | Gansu | - | - | - | - | - | - | - | - | - | - | - | - | - | - | - | + | + | - | - | + | - | - | + | - | - | + | + | + | + | + | + | + | + | + | + | + |
| 21-D-2 | goat | Gansu | - | - | - | - | - | - | - | - | - | - | - | - | - | - | - | + | + | - | - | + | - | - | + | - | - | + | + | + | + | + | + | + | + | + | + | + |
| 21-D-3 | goat | Gansu | - | - | - | - | - | - | - | - | - | - | - | - | - | - | - | + | + | - | - | + | - | - | + | - | - | + | + | + | + | + | + | + | - | + | + | + |
| 21-D-4 | goat | Gansu | - | - | - | - | - | - | - | - | - | - | - | - | - | - | - | + | + | - | - | + | - | - | + | - | - | + | + | + | + | + | + | + | - | + | + | + |
| 21-D-5 | goat | Gansu | + | - | + | + | + | + | + | + | - | - | - | - | - | - | - | + | + | - | - | + | - | + | + | - | - | + | + | + | + | + | + | + | + | + | + | + |
| N16 | cow | Gansu | + | - | - | - | - | - | - | + | - | - | - | - | - | - | - | + | + | - | - | + | - | - | - | - | - | + | + | + | + | + | + | + | + | + | + | + |
| N35 | cow | Gansu | + | - | - | - | - | - | - | - | - | - | - | - | - | - | - | + | + | - | - | + | - | - | - | - | - | + | + | + | + | + | + | + | + | + | + | + |
| N40 | cow | Gansu | - | - | - | - | - | - | - | - | - | - | - | - | - | - | - | + | + | - | - | + | - | - | - | - | - | + | + | + | + | + | + | + | + | + | + | + |
| N47 | cow | Gansu | - | - | - | - | - | - | - | - | - | - | - | - | - | - | - | + | + | - | - | + | - | - | - | - | - | + | + | + | + | + | + | + | + | + | + | + |
| C10 | chicken | Shanxi | - | + | - | - | - | - | - | - | + | + | - | - | - | - | - | - | + | - | - | + | - | - | - | - | - | + | + | + | + | - | - | - | - | - | + | + |
| C17 | chicken | Shanxi | - | + | - | - | - | - | - | - | + | + | - | - | - | - | - | - | + | - | + | + | - | - | - | - | + | + | + | + | + | - | + | + | + | + | + | + |
| C28 | chicken | Shanxi | + | - | - | - | - | - | - | - | - | - | - | - | - | - | - | + | + | - | - | + | - | - | - | - | - | + | - | + | + | - | - | - | - | - | - | + |
| C30 | chicken | Shanxi | + | - | - | - | - | - | - | - | - | - | - | - | - | - | - | + | + | - | - | + | - | - | - | - | - | + | + | + | + | + | + | + | + | + | + | + |

TS= Tibetan sheep; -: negative; +: positive
